# Immunotherapy of Head and Neck Cancer: Current and Future Considerations

**DOI:** 10.1155/2009/346345

**Published:** 2009-08-09

**Authors:** Alexander D. Rapidis, Gregory T. Wolf

**Affiliations:** ^1^Department of Head and Neck Surgery, Greek Anticancer Institute, Saint Savvas Hospital, 171 Alexandras Avenue, 115 22 Athens, Greece; ^2^Department of Otolaryngology Head and Neck Surgery, A. Alfred Taubman Health Care Center, University of Michigan Health System, Ann Arbor, MI 48109, USA

## Abstract

Patients with head and neck squamous cell carcinoma (HNSCC) are at considerable risk for death, with 5-year relative survival rates of approximately 60%. The profound multifaceted deficiencies in cell-mediated immunity that persist in most patients after treatment may be related to the high rates of treatment failure and second primary malignancies. Radiotherapy and chemoradiotherapy commonly have severe acute and long-term side effects on immune responses. The development of immunotherapies reflects growing awareness that certain immune system deficiencies specific to HNSCC and some other cancers may contribute to the poor long-term outcomes. Systemic cell-mediated immunotherapy is intended to activate the entire immune system and mount a systemic and/or locoregional antitumor response. The delivery of cytokines, either by single cytokines, for example, interleukin-2, interleukin-12, interferon-*γ*, interferon-*α*, or by a biologic mix of multiple cytokines, such as IRX-2, may result in tumor rejection and durable immune responses. Targeted immunotherapy makes use of monoclonal antibodies or vaccines. All immunotherapies for HNSCC except cetuximab remain investigational, but a number of agents whose efficacy and tolerability are promising have entered phase 2 or phase 3 development.

## 1. Introduction

Head and neck cancer is a prevalent condition in the United States and the eighth leading site of new cancer cases among men. It is estimated that 35,310 new cancers of the oral cavity and pharynx will have been diagnosed in 2008 in the United States, and that 7,590 Americans will have died due to such cancers [1]. More than 80% of head and neck cancers (excluding cancers of the thyroid, salivary glands, and nasopharynx; and nonmelanoma skin cancer) are head and neck squamous cell carcinomas (HNSCCs) [2].

Death rates from cancers of the oral cavity and pharynx declined from 1979 to 2000 in the United States, but they have since then remained stable. The overall 5-year relative survival rate at diagnosis is 59.1%, with a range from 81.8% for early disease at diagnosis to 26.5% for advanced disease. For diagnoses at all stages combined, the 10-year relative survival rate for cancers of the oral cavity and pharynx is 48%. For cancer of the larynx, the 5-year relative survival rate at all stages of diagnosis is 62.9%, ranging from 81.1% for early cancers to a dismal 23.9% for cancers with distant metastases at the time of diagnosis [1]. 

Mortality in head and neck cancers in the United States is higher in blacks than in whites: for cancer of the larynx, the 5-year survival rate in 2000 was 67% for whites and 40% for blacks; for cancer of oral cavity and pharynx, the rate was 65% for whites and 46% for blacks [3]. 

In light of these discouraging data, the development of novel therapies for HNSCC has become a priority. One of the most exciting research avenues is immunotherapy, thanks to advances in the understanding of the relationships between tumors and the host immune system, as well as to developments in the technology for identifying molecular therapeutic targets. This article reviews the rationale for immunotherapy in HNSCC and the principal approaches under investigation.

## 2. Etiology, Diagnosis, and Staging of HNSCC

The development and progression of HNSCC are considered to result from stepwise alterations of cellular, genetic, biochemical, and molecular pathways at multiple epithelial sites within the aerodigestive tract [4]. This progression probably explains, in part, the high incidence of second primary tumors, the tendency for patients to present with premalignant lesions at multiple sites in the aerodigestive tract, and the high rate of progression of these premalignancies [5]. 

Tumor carcinogenesis in HNSCC involves dynamic interactions among many factors. Exposure of the upper aerodigestive tract to alcohol or tobacco is one of the chief risk factors for many HNSCCs, and exposure to both increases the risk beyond what would be expected if the agents simply had additive effects [2]. Another common risk factor is alteration of the function of the p53 tumor suppressor gene, which may be caused by either gene mutation or infection with an oncogenic type of human papillomavirus (HPV) [6, 7]. In some patients, particularly those with oropharyngeal cancer not associated with p53 mutation or the molecular impacts of alcohol and tobacco, HPV infection can cause head and neck cancer even in the absence of other molecular alterations [4, 8]. All of these risk factors are likely to result from and contribute to suppression of the patient's immune system, as is the tumor itself [9]. 

Diagnosis of HNSCC is based on a history and physical examination and computed tomography and/or magnetic resonance imaging as needed, chest imaging, pathology review, and biopsy [10]. In advanced HNSCC, positron emission tomography is an increasingly useful new modality for assessing lymph node involvement, distant metastases, and synchronous second primary tumors [11]. 

Relatively small primary HNSCCs with no nodal involvement are usually classified as stage I or II, and large primary tumors that may have invaded nearby structures or spread to regional lymph nodes are classified as stage III or IV [10]. Generally, stage I or II disease is discussed as “early stage” and stage III or IV disease is termed “advanced stage” [12].

## 3. Current Therapeutic Options

Approximately 40% of patients with HNSCC present with early-stage disease, and either surgical resection or radiotherapy is recommended as a single treatment modality [10]. Most patients (60%) present with locally advanced disease [10] and require a multidisciplinary approach using some combination of surgery, radiotherapy, and chemotherapy [4, 13].

In addition to considering the stage of cancer, oncologists must equally consider the site of disease. Lesions in the oral cavity are often treated with surgery followed by radiotherapy or chemoradiotherapy (CRT). Tumors located in the oropharynx, hypopharynx, nasopharynx, or larynx are usually treated with CRT firs [14].

As it is in many other kinds of cancer, immunotherapy is emerging as an important new option in treating HNSCC. The monoclonal antibody (MAb) cetuximab, which binds to the EGF receptor, is approved by the US Food and Drug Administration as first-line treatment of locally or regionally advanced HNSCC in combination with radiotherapy. As a single agent, cetuximab is indicated for the treatment of patients with recurrent or metastatic HNSCC for whom prior platinum-based therapy has failed [15]. Although technically considered a molecular targeted agent that inhibits the EGFR, cetuximab is a chimeric MAb. Its administration is often associated with a generalized allergic skin rash that correlates directly with tumor responses. Whether the benefit of adding this agent is due to EGFR inhibition and downstream molecular effects on pathways of cell proliferation and apoptosis or due to antibody-mediated immune responses is unclear. It is less likely due to a direct allergic response since nonneutralizing antibodies to cetuximab are only detected in 5% of treated patients. Recent evidence has shown that MAbs mediate antibody-dependent cellular cytotoxicity, and induce activation of cellular immunity, including natural killer and T cells [16]. Other immunotherapies being explored for treatment of HNSCC are discussed later in this paper.

In recent years, there have been many improvements in the modalities used to treat HNSCC. Minimally invasive surgical techniques followed by improved reconstruction procedures frequently result in better functional and esthetic outcomes. Improved microvascular reconstructions have enhanced functional results of major tumor resections. Intensity modulation in the use of radiation therapy may be reducing toxicity, and altered fractionation schedules may be improving local disease control and late toxicity [4]. Multidrug chemotherapy regimens incorporating the newest agents and molecular targeted therapies have shown some efficacy and tolerable toxicity in both recurrent and previously untreated patients.

In addition to efficacy considerations, impact on quality of life remains a major consideration in selecting appropriate treatment for HNSCC. The tumors themselves commonly jeopardize physiologic functions, such as the patient's ability to chew, breath, and swallow; the senses of taste, smell, and/or hearing; as well as personal characteristics such as voice and appearance [10]. Common side effects of radiotherapy include fibrosis of normal tissue, scarring, and long-term dry mouth or dysphagia [17]. CRT involves the substantial risks for severe acute and long-term side effects associated with both chemotherapy and radiotherapy, including mucositis, dermatitis, pain, dysphagia, dry mouth, local, or systemic infections, dental problems, depression, speech difficulties, and occasionally breathing difficulties, as well as immune suppression [14]. Combination with altered fractionation or intensified radiation increases the burden. Cisplatin, the preferred chemotherapeutic agent in CRT [10], is severely toxic when used in combination with other drugs and radiation, and patients unable to tolerate cisplatin have an especially high cumulative risk of death: 20% to 25% at 2 years [14].

## 4. Novel Therapeutic Directions: Engaging the Immune System and Antitumor Immunity

The HNSCC patient's immune system is an important element in the development of the disease and, in many cases, in the response to treatment. The microenvironment in which HNSCC arises is populated with numerous immune cells and soluble factors produced by these cells. Both cutaneous skin and aerodigestive tract mucosa are highly immunoreactive organs. In this environment, it is likely that many newly appearing tumor cells will be rapidly eliminated, leaving those which survive particularly resistant to the body's innate and adaptive immune mechanisms [18].

As with other cancers, there are numerous methods by which HNSCC may avoid recognition and destruction by the immune system. One strategy is to escape immune system recognition via downregulation of human leukocyte antigens (HLAs), which are necessary to present antigens on malignant cells to T cells [19, 20], or via apoptosis of circulating T cells, which seems to be mediated at least in part by tumor-derived Fas ligand [21]. Another potential mechanism is secretion of immunosuppressive factors such as prostaglandin E_2_ [22], vascular endothelial growth factor (VEGF) [23], interleukin (IL)-10, or transforming growth factor-*β* [24]. Additionally, immune defenses can be directly inhibited by “suppressor T cells,” now known as regulatory T cells (T_reg_) [9]. Immune reactivity is not simply turned on or off, rather, HNSCC and certain other cancers avoid the immune response by modulating responses that are more effective against tumors, for example, T_H_1 responses, and enhancing those which are less effective, for example, T_H_2 responses. T_H_1 responses are classically defined by the production of interferon (IFN)-*α*, granulocyte macrophage colony-stimulating factor (GM-CSF), and IL-2, whereas T_H_2 responses are defined by expression of cytokines such as IL-4, IL-6, and IL-10 [24]. 

In most types of cancer, these processes are thought to take place concurrently [24]. Some immune system deficiencies, however, are specific to HNSCC and a few other cancers [25], and they are thought to contribute to the poor long-term survival rate in HNSCC. Patients with HNSCC have been shown to have lymph nodes that are reduced in size and have diminished T-cell content. Reduced T-cell function has been linked to shorter disease-specific survival [26]. Defects in dendritic cell (DC) function are also a hallmark of immune system dysfunction in HNSCC [27]. For example, the accumulation of histiocytes/DCs in the distended sinuses of lymph nodes, known as sinus histiocytosis, is a reflection of DC defects, and is present in the lymph nodes of HSNCC patients. The buildup of these cells in the nodal sinuses prevents their entry into the node parenchyma, and maturation is, therefore, impaired, preventing optimal T-cell stimulation [28]. Low infiltration of DCs in tumor environments (linked to abnormalities in the TcR-associated zeta chain in TILs) was correlated with poor prognosis for disease survival [29]. 

Specific defects in cell-mediated immunity may also include progressive decreases in dermal delayed-type hypersensitivity responses, T-cell counts in blood, proliferative responses of blood T cells to mitogens or antigen stimulation, and blood monocyte functions such as chemotaxis and cytotoxicity [25]. One example is the production by HNSCC and some other cancers of chemoattractive factors (e.g., VEGF) to attract immunosuppressive CD34(+) progenitor cells that inhibit the capacity of intratumoral lymphoid cells to become activated [23, 30]. Intriguingly, cell-mediated immunity may decline even before the tumor develops, whereas levels of B cells in blood, immunoglobulin, and complement are usually normal. Therefore, alterations in humoral immunity seem modest in HNSCC patients [25]. These findings reflect the fact that HNSCC is intrinsically characterized by deficits in the cellular immune system. These cancers arise within the oral, nasal, or laryngeal mucosa, and interact with the local, regional, and systemic immune cells likely to affect the initiation and promotion of tumors in these environments [18].

## 5. Immunotherapy: Future Directions for HNSCC Treatment

Immunotherapy is an attractive option for cancer treatment because both humoral immunity and cell-mediated immunity involve cells with a variety of clonally distributed antigen receptors that can distinguish normal cells from cancerous cells. Another advantage is that the immune system can adapt to the evolution of cancer cells and can respond in a systemic fashion [31]. Signs of an immune response have been shown to correlate with positive outcomes for cancer patients. For example, the presence of tumor-infiltrating T cells has been correlated with progression-free survival and/or overall survival in various cancers, including advanced ovarian cancer [32], advanced melanoma [33], and head and neck cancer [34]. Because the immunobiology of HNSCC is so intimately associated with the host immune system, the reversal of immunosuppression is a particularly attractive therapeutic goal in this tumor type [18]. The remainder of this article describes immunotherapies now in development for treatment of HNSCC.

### 5.1. Systemic Cell-Mediated Immunotherapy in HNSCC

Systemic cell-mediated immunotherapies are nonspecific, and attempt to replace the entire immune system by mounting either a systemic and/or locoregional antitumor response. For example, adoptive transfer therapy is a form of passive therapy that entails ex vivo expansion and modification of the patient's own immune cells, followed by their reinfusion. The initial use of this approach was based on evidence from murine studies in which regression of established tumors was demonstrated [18]. An example of its clinical application was shown in patients with stage IV nasopharyngeal carcinoma, which expresses Epstein Barr virus (EBV) antigens. EBV-specific autologous T cells were reactivated and expanded exogenously from peripheral blood lymphocytes by stimulating them with EBV-transformed autologous B cells. Aside from mild inflammatory reactions in 2 patients, treatment was well tolerated, and 6 of 10 patients demonstrated control of disease progression [35]. Other groups have reported the feasibility of generating tumor-reactive T cells and the low toxicity of this approach in advanced HNSCC [36, 37]. 

In transfected dendritic cell therapy, autologous dendritic cells are transfected with patient tumor DNA, then reinfused. A proof-of-concept study in HNSCC showed that this approach yielded effective antigen-presenting cells, without signs of tumor-induced suppression of dendritic cells [38]. Another novel approach is the use of intratumoral dendritic cells in combination with immunosuppressive chemoradiation. Augmentation of immune responses, long-term tumor regressions, and increased apoptosis associated with decreases in intratumoral regulatory T cells have recently been shown in an animal model of head and neck cancer [39]. 

Cytokine-based immunotherapy works by delivering proinflammatory cytokines either locoregionally and/or systemically to elicit an antitumor response. A number of cytokines are being explored for treatment of HNSCC, including GM-CSF, IL-2, IFN-*γ*, IL-12, and an investigational multicytokine biologic known as IRX-2. Table 1  lists the approaches to systemic cell-mediated immunotherapy for HNSCC that are currently in clinical trials [40], of which some are discussed in more detail in what follows.

**OncoVEX^GM-CSF^**

OncoVEX^GM-CSF^ is a second-generation oncolytic herpes simplex virus that delivers GM-CSF. In a phase 1 trial, multiple doses of OncoVEX^GM-CSF^ were safe and well tolerated in patients with a range of solid tumor types, GM-CSF was expressed, and there was evidence of antitumor activity [41]. According to preliminary data from a phase 1/2 study specific to node-positive advanced head and neck cancer, the combination of CRT and OncoVEX^GM-CSF^ produced pathologic complete response in 6 of 8 patients, and non-CRT-related toxicities were mild [42].

**Interleukin-2**

The main function of IL-2, one of the major proinflammatory cytokines produced by T cells, is to enhance the growth and cytotoxic response of activated T cells [43]. Multiple studies have shown that IL-2 enhances cellular immune responses to tumors by stimulating the proliferation and activation of several types of leukocytes with antitumor activity, including natural killer cells, lymphokine-activated killer cells, antigen-specific T-helper cells, cytotoxic lymphocytes, macrophages, and B cells [44]. The nonspecific immune reaction first causes tumor shrinkage, followed by tumor-specific, delayed-type hypersensitivity, and long-lasting immune memory [45]. Complete or partial responses have been reported after IL-2 or IL-2-based immunotherapy in head and neck cancer patients [43]. IL-2 has been administered to HNSCC patients using a variety of delivery methods, including intralesional injection (recombinant IL-2) and synthetic gene delivery systems.In addition to the benefits of IL-2 itself, the attributes of some delivery methods may have immunologically beneficial effects, whereas other methods, such as viral-based vectors, can increase toxicity. In a murine model, giving IL-2 in a plasmid/cationic lipid formulation resulted not only in expression of the IL-2 transgene but also in induction of endogenous IFN-*γ* and IL-12 [44].Several novel methods of administering IL-2 have been investigated, including direct administration of low-dose recombinant IL-2 around the chin and neck lymph nodes in HNSCC patients [45].

**Interferon-*γ***

Interferon-*γ* has not been well studied in HNSCC, but systemic administration of the recombinant form in a phase 1/2 study in 8 patients produced clinically measurable immunologic responses in 4 of 9 HNSCC tumors evaluated, resulting in clinically measurable response in 3 patients and stable disease in 4 (1 patient progressed). During 22 days of treatment, a carcinoma in situ in the piriform sinus disappeared, and the other 3 tumors were reduced in bulk by 40%, 40%, and 18% [46].

**Interferon-*α***

Interferon-*α* has been added to other drugs in the treatment of HNSCC. The combination of IFN-*α*, cisplatin, and 5-fluorouracil was associated with an overall response rate of 55% in patients with advanced esophageal cancer, accompanied by considerable toxicity [47]. In a phase 2 study of interferon-*α* plus isotretinoin and vitamin E in patients with locally advanced HNSCC, the 5-year progression-free survival rate was 80% and the 5-year overall survival rate was 81.3% [48]. Combination treatment with low dose recombinant IL-2 and interferon alpha-2a has also produced significant clinical tumor regressions in 2 of 11 (18%) heavily pretreated patients with recurrent disease [49].

**Interleukin-12**

Interleukin-12 has effects on both the innate and adaptive immune systems. It is important in inducing cellular immunity because it fuels the production and activation of cytolytic T cells and natural killer cells and induces the production of cytokines. In a study of 30 patients with previously untreated HNSCC, injection of recombinant IL-12 into the primary tumor was shown to increase the number of natural killer cells and alter the distribution of B cells in the lymph nodes of the 10 treated patients. These effects included redistribution of lymphocytes from the peripheral blood to the lymph nodes in the neck; a significant increase in natural killer cells and a lower percentage of T_H_cells in the lymph nodes and the primary tumor; and a 128-fold increase in IFN-*γ* mRNA in the lymph nodes. Finally, the T_H_2 profile in the lymph nodes of IL-12-treated patients switched to a T_H_1 profile [50].

**IRX-2**

IRX-2 is a promising systemic cell-based strategy for HNSCC immunotherapy that employs a multifaceted approach to stimulating immune response. A primary cell-derived biologic IRX-2 contains multiple cytokines: IL-1, -2, -6, and -8, tumor necrosis factor-*α*, IFN-*γ*, G-CSF, and GM-CSF.It is sterile, endotoxin-free, and serum-free, and is produced from purified human mononuclear cells that are stimulated by phytohemagglutinin (PHA) under GMP conditions [51]. Additionally, in the regimen, cyclophosphamide is used to inhibit suppressor T-cell function, indomethacin is used to block immunosuppression due to the prostaglandins synthesized by the tumor and by suppressor macrophages, and zinc is used to reverse cellular immunodeficiency [52].

It has been shown that ex vivo treatment with IRX-2 leads to dose- and time-dependent apoptosis suppression of T cells (*P* < .001 to *P* < .005). IRX-2 also potentiated antitumor effects of immune cells, such as upregulation of key signaling molecules' expression on dendritic cells to increase their functions. Local delivery of IRX-2 induced systemic changes in both peripheral blood memory and naive T cell subsets [53].

The results of a multicenter phase 2 trial of IRX-2 have recently been reported. In this trial, 27 previously untreated, resectable patients with stage II-IV oral cavity (15), oropharynx (8), larynx (3), or hypopharynx (1) HNSCC received the IRX-2 regimen prior to surgery. The regimen consisted of intravenous cyclophosphamide on day 1, followed by bilateral perilymphatic injections of IRX-2 (115 U bilateral daily) from day 4 to 15, and daily oral indomethacin, zinc, and omeprazole from day 1 to 21. The IRX regimen was well tolerated, with minimal acute toxicity (grade <2). Tumor responses (>12% decrease on blinded CT review) were seen in 16% of patients, and 74% patients had either reduction or stable tumor size. Significant changes in tumor and lymph node lymphocytic infiltration were observed in the IRX-treated patients. Data on estimated 2-year overall survival (72%) and disease-free survival (67%) were favorable compared to those reported for 81 concurrent treatment matched controls [54, 55].

### 5.2. Targeted Immunotherapy in HNSCC

Technological advances have allowed researchers to identify several kinds of tumor-associated antigens that are now under investigation as therapeutic targets in HNSCC [56]. One category is tumor-specific antigens (also called germ cell antigens or cancer testes antigens), which are silenced in normal tissues but are reactivated in certain tumors [31]. For example, up to 71% of HNSCCs express antigens from at least 1 of 6 melanoma antigen genes (MAGEs) [57], notably MAGE-1 and MAGE-3 [58]. Antigen from NY-ESO-1, a gene expressed in normal ovary and testis, is highly expressed in a variety of tumor types [59], including HNSCC [60]. 

Another category of tumor-associated antigen is tumor-specific mutated proteins that are unique to the tumor and may contribute to the malignant phenotype, for example, tumor suppressor gene p53 [31]. Preclinical work suggests not only that p53 is mutated in many more cases of HNSCC than originally thought but also that wild-type p53 is often associated with highly oncogenic strains of HPV (types 16 and 18) [61]. 

Antigens overexpressed in tumors are a third category of targets under investigation. Notable examples are carcinoembryonic antigen (CEA), HER-2/neu, VEGF, and EGFR [31]. Antigens derived from oncogenic viruses, such as the HPV E6 and E7 oncoproteins, are also important targets in HNSCC [62–64]. 

#### 5.2.1. Monoclonal Antibody (MAb) Immunotherapy

Advancements in technology have allowed identification and large-scale production of monoclonal antibodies, which are highly specific to their target, are better tolerated than cytotoxic drugs, and can induce tumor cell apoptosis [31]. These advantages have made MAb immunotherapy a compelling field of research (Table 2) [40]. 

EGFR is overexpressed in more than 90% of HNSCCs [10], and overexpression is often associated with poor clinical prognosis and outcome, including reduced disease-free and overall survival. A variety of EGFR inhibitors have been developed that function either by binding to the extracellular ligand binding domain of the EGF receptor (e.g., MAbs such as cetuximab), or by inhibiting the intracellular tyrosine kinase activity of the receptor [65, 66]. While the exact mechanisms of action of these inhibitors are unclear, cetuximab has been shown to activate antibody-dependant cellular cytotoxicity (ADCC). The in vivo success of cetuximab in combination with radiation has inspired exploration of other anti-EGFR agents. These include matuzumab [67], panitumumab (also called ABX-EGF) [68], ICR62 [69], nimotuzumab (also called h-R3) [70], MAb 806 [71], and zalutumumab [66]. Anti-EGFR agents have recently been reviewed elsewhere [66]. 

VEGF is highly expressed in most human cancers [72, 73], and in HNSCC its expression may be a significant factor in survival [74]. Therefore, recent studies have combined the antiangiogenic agent bevacizumab with chemotherapy. For example, an ongoing phase 2 trial (*N* = 14) pairs bevacizumab with pemetrexed in first-line treatment of recurrent and/or metastatic HNSCC; interim results show an overall response rate of 45% among the 11 evaluable patients, but also a high rate of bleeding complications in susceptible patients [75]. Bevacizumab is also being investigated in head and neck cancer in combination with erlotinib, a small-molecular-weight tyrosine kinase inhibitor [76]. 

There is evidence that VEGF and VEGF receptor-2 are coexpressed in HNSCC and that coexpression is associated with a higher proliferation rate and worse survival [74]. Adjuvant therapy with VEGFR-2 inhibitors might disrupt both the paracrine and autocrine actions of VEGF and be beneficial in HNSCC patients [77]. 

An investigational anti-VEGF antibody, 2C3, appears to control tumor metastasis by a mechanism somewhat different from that of bevacizumab: in a preclinical study of breast cancer, it inhibited lymphangiogenesis and decreased intratumoral lymph vessel development [78].

Another new treatment strategy is to target CEA, an antigen present on the surface of a majority of HNSCC tumors [31, 79], via MAb immunotherapy plus radiotherapy. A phase 1 trial combined high-dose labetuzumab, a ^90^Y-labeled humanized anti-CEA MAb, with doxorubicin and peripheral blood stem cell rescue for patients with advanced thyroid cancer. Objective responses were rare, but the therapy was well tolerated and there was evidence of antitumor activity [80]. Another study in advanced thyroid cancer evaluated bispecific MAb (BsMAb), which targets both CEA and diethylenetriamine penta-acetic acid. Combination therapy with BsMAB and a ^131^I-labeled bivalent hapten was associated with a median survival time of 110 months, significantly longer than the 61 months seen in untreated patients (*P* < .03) [81].

#### 5.2.2. Cancer Vaccines

Two common types of therapeutic cancer vaccines are peptide/protein-based or dendritic cell-based. To produce the first type, an adjuvant is combined with 1 or more peptides/proteins commonly expressed on HNSCC such as p53, MAGE, or HPV. It is expected that the immune system, in response to the adjuvant, will also respond to tumor cells that express the antigen(s). For the second type, dendritic cells are removed from cancer patients through leucopheresis and stimulated with an appropriate tumor antigen, then reinjected so that they will activate T cells specific to the patient's tumor. The strategies can be combined, as when dendritic cells are pulsed with mutant p53 peptides [82, 83]. A phase 1 trial of this approach is under way [40]. Dendritic cells can also be pulsed with MAGE peptides. In one recent study, a vaccine that combines MAGE-1 and MAGE-3 peptides was administered following surgery and chemotherapy for 2 patients with primary malignant melanoma of the esophagus, which has an extremely poor prognosis. One patient had stable disease for 5 months and survived for 12; the second was without tumor recurrence for 16 months after treatment, and, following esophagectomy, had survived for 49 months at the time of trial report publication [84]. In a phase 1/2 study, an MAGE-3 peptide + ASO2B adjuvant vaccine produced clinical responses in 6 of 12 patients with metastatic tumors (mainly melanoma), but the response could not be clearly correlated with cytokine profile, levels of anti-MAGE-3 antibody, or IgG subclass [85]. A phase 2 pilot study has recently been completed that made use of vaccines constructed of HPV 16 peptides E6 and E7 alone or in combination with MAGE-3 peptides [40]. A common issue challenging the further development of clinically useful vaccines is the need to develop new and more effective vaccine adjuvants. 

Cancer vaccines for HNSCC can also be based on DNA or RNA. The nucleic acid containing the gene for the antigen is manipulated exogenously so it will be taken up, expressed, and processed by antigen-presenting cells, in the hope that the immune system will target tumor cells containing the same antigen. Vaccines of this type have shown potential for targeting CEA when recombinant fowlpox or ALVAC (canarypox) viruses, which do not replicate in human cells, are used as vectors, with and without GM-CSF [86, 87]. Nucleotide-based vaccines targeting HPV are also being studied in HNSCC. Research in China showed that in a mouse model of esophageal SCC, a fusion protein vaccine combining the HPV-16 oncoproteins E6 and E7 significantly inhibited tumor growth and size (*P* < .01), and 25% of vaccinated animals remained tumor-free at 2 days [88]. In another study, Chen et al. constructed a vaccine which linked *Mycobacterium tuberculosis* heat-shock protein 70 to HPV-16 E7; the E7-specific T-cell response to murine tumors that expressed HPV-16 E7 was at least 30-fold higher with the fusion vaccine than with a vaccine based on unmodified E7 [59]. A list of cancer vaccines in clinical trials for treatment of head and neck cancer is provided in Table 3  [40].

## 6. Conclusions

Given the well-established role of immune system dysfunction in HNSCC, immunotherapy is an attractive treatment option, potentially associated with more tolerable side effects and improved efficacy. Recent advances in identifying HNSCC tumor antigens have provided targets for monoclonal antibodies and other modes of immunotherapy. In particular, advances in the understanding of cell-mediated immunity have led to several promising approaches to HNSCC treatment that involve systemic cell-mediated immunotherapy, such as the delivery of cytokines that can stimulate a durable immune response and tumor rejection. These novel treatment modalities, either as monotherapy or combined with other forms (e.g., MAb therapy), represent future directions in the treatment of HNSCC.

## Figures and Tables

**Table 1:** Systemic cell-mediated immunotherapies in clinical development in head and neck cancer [40].

Agent	Phase	Status	Study Type	Description
IFN-*α*	2 (NCT00004897)	Active, not recruiting (*N* ~ 15–45)	Open-label trial	Patients with stage I–III esophageal cancer receive combination chemotherapy and recombinant IFN-*α* followed by surgery and/or RT
	3 (NCT00054561)	Completed (*N* = 376)	Multicenter randomized controlled trial	To compare the combination of isotretinoin, recombinant IFN-*α*, and vitamin E with observation only in patients with stage III or IV HNSCC

Pegylated IFN-*α*2b	2 (NCT00276523)	Completed (*N* = 72)	Randomized controlled trial	Pegylated IFN-*α*2b at 3 different dose levels is compared with no treatment prior to resection of stage II–IV HNSCC

IL-2	2 (NCT00006033)	Completed (*N* = 80)	Multicenter open-label	To compare IL-2 gene with methotrexate in the treatment of recurrent or refractory stage III/IV HNSCC
	3 (NCT00002702)	Recruiting (*N* ~ 260)	Multicenter randomized, controlled trial	To compare surgery and RT with and without rIL-2 in patients with SCC of the mouth or oropharynx

IL-12	1/2 (NCT00004070)	Active, not recruiting (*N* ~ 28–34)	Multicenter rising-dose study	Patients with unresectable, recurrent, or refractory HNSCC receive IL-12 gene twice during week 1 and once weekly during weeks 2–7

ALT-801 (a recombinant fusion protein with an IL-2 component)	1 (NCT00496860)	Recruiting (*N* ~ 46)	Multicenter dose-escalation study	To determine the MTD of ALT-801 in previously treated patients with progressive metastatic malignancies, including HNC

IRX-2	2 (NCT00210470)	Closed (*N* = 27)	Multicenter open-label trial	Study of IRX-2 with cyclophosphamide, indomethacin, and zinc in patients with newly diagnosed, resectable stage II–IV HNSCC. The study is being conducted to confirm the safety and biological effect of the IRX-2 regimen in the same population to be studied in a planned randomized phase 3 trial. The primary focus will be on observations made from the start of treatment through the planned surgical resection of the primary tumor.

HNC: head and neck cancer; HNSCC: head and neck squamous cell carcinoma; IFN: interferon; IL: interleukin; MTD: maximum tolerated dose; RT: radiotherapy.

**Table 2:** Monoclonal antibodies (excluding anti-EGFR agents) in clinical development in head and neck cancer [40].

Agent	Phase	Status	Study Type	Description
Bevacizumab	Clinicaltrials.gov search retrieves records for 3 phase 1 trials 2 phase 1/2 trials 11 phase 2 trials 1 phase 3 trial	The phase 1, 1/2, and 2 trials are completed or ongoing The phase 3 trial is recruiting	Several	The early-phase trials are exploring several different regimens The phase 3 trial is a multicenter, randomized, controlled trial in which patients with recurrent or metastatic HNSCC receive chemotherapy ± bevacizumab. Chemotherapy consists of cisplatin, docetaxel, and fluorouracil.

anti-CD45 MAb	1 (NCT00608257)	Completed (*N* = 18)	Dose-escalation study	Patients with EBV-positive nasopharyngeal cancer receive autologous EBV-specific cytotoxic T cells in combination with anti-CD45 MAb

MN-14 (anti-CEA MAb)	1/2 (NCT00004048)	Active, not recruiting(*N* ~ 30)	Dose-escalation study	Patients with medullary thyroid cancer undergo radioimmunotherapy with MN-14 alone or combined with doxorubicin and peripheral blood stem cell rescue

CEA: carcinoembryonic antigen; EBV: Epstein-Barr virus; HNSCC: head and neck squamous cell carcinoma; MAb: monoclonal antibody.

**Table 3:** Vaccines in clinical development in head and neck cancer [40].

Agent	Phase	Status	Study Type	Description
ALVAC-CEA vaccine	2 (NCT00003125)	Active, not recruiting (*N* ~ 24)	Partially randomized pilot study	For patients with CEA-expressing advanced tumors, including HNC. In stage I, patients receive vaccinia-CEA vaccine and then ALVAC-CEA (CEA-avipox) vaccine, or the reverse sequence. In stage 2, patients receive whichever vaccine was superior, plus GM-CSF ± IL-2.

Anti-CEA RNA-pulsed DC vaccine	1 (NCT00004604)	Active, not recruiting (*N* ~ 18)	Dose-escalation study	To determine the MTD of the vaccine in patients who have refractory metastatic cancer, including HNC, that expresses CEA

EBV LMP-2 peptide vaccine	1 (NCT00078494)	Completed (*N* = 99)	Randomized study	Patients with nasopharyngeal cancer that has been controlled with standard therapy receive 1 of 2 LMP-2 vaccines to determine which better prevents cancer recurrence. LMP-2 is a protein produced by EBV.

HPV-16 E7/E6 peptide vaccine	1 (NCT00019110)	Completed (*N* = 40–46)	Multicenter open-label study	Patients with advanced or recurrent cancers, including HNC, receive a vaccine that contains the HPV-16 E7 and E6 peptides

JAX-594 (thymidine kinase-deleted vaccinia virus plus GM-CSF)	1 (NCT00625456)	Recruiting (*N* ~ 24)	Dose-escalation study	To find the MTD of JAX 594 in patients with refractory solid tumors, including HNSCC

MAGE-A3/HPV-16 vaccine	1 (NCT00257738)	Recruiting (*N* ~ 90)	Dose-escalation study	Patients with HNSCC receive a vaccine comprised of MAGE-A3 and HPV-16 peptides
	1 (NCT00704041)	Recruiting (*N* ~ 48)	Dose-escalation study	To evaluate 4 doses of the MAGE-A3/HPV-16 vaccine in 2 cohorts of HNSCC patients those with MAGE-A3-positive tumors and those with HPV-16-positive tumors

Multiple-peptide vaccine (LY6K, VEGFR1, VEGFR2)	1 (NCT00561275)	Completed (*N* = 6)	Open-label trial	Patients with esophageal cancer receive a vaccine containing multiple peptides and GM-CSF

p53-pulsed DC vaccine	1 (NCT00404339)	Recruiting (*N* ~ 50)	Randomized safety trial	Patients with HNSCC receive autologous DCs loaded with wild-type p53 peptides, ± T-helper peptide epitope

Ras peptide vaccine	2 (NCT00019331)	Completed (*N* = 60)	Single-center trial	To compare 3 regimens of vaccine therapy with tumor-specific mutated Ras peptides plus IL-2 or GM-CSF in patients with metastatic solid tumors, including HNC, that potentially express mutant Ras.

Fowlpox-CEA-TRICOM vaccine (fCEA-TRI)	1 (NCT00028496)	Completed (*N* = 48)	Dose-escalation study	To evaluate fCEA-TRI ± GM-CSF in patients with advanced or metastatic cancer, including HNC.
	1 (NCT00021424)	Completed (*N* = 20)	Dose-escalation study	To find the MTD of fCEA-TRI in patients with advanced SCC of the oral cavity or oropharynx or nodal or dermal metastases
	1 (NCT00027534)	Completed (*N* = 6–18)	Dose-escalation study	Immunotherapy comprises autologous DCs treated with fCEA-TRI in patients with CEA-expressing advanced or metastatic cancer, including HNC.

CEA: carcinoembryonic antigen; DC: dendritic cell; EBV: Epstein-Barr virus; HNC: head and neck cancer; HNSCC: head and neck squamous cell carcinoma; IL: interleukin; GM-CSF: granulocyte macrophage colony-stimulating factor; HPV: human papillomavirus; LY6K: lymphocyte antigen 6 complex, locus K; MAGE: melanoma antigene gene; MTD: maximum tolerated dose; TRICOM: TRIad of COstimulatory Molecules (aimed at stimulating a cytotoxic T-cell response); VEGFR: vascular endothelial growth factor receptor.
